# Building Automation Pipeline for Diagnostic Classification of Sporadic Odontogenic Keratocysts and Non-Keratocysts Using Whole-Slide Images

**DOI:** 10.3390/diagnostics13213384

**Published:** 2023-11-04

**Authors:** Samahit Mohanty, Divya B. Shivanna, Roopa S. Rao, Madhusudan Astekar, Chetana Chandrashekar, Raghu Radhakrishnan, Shylaja Sanjeevareddygari, Vijayalakshmi Kotrashetti, Prashant Kumar

**Affiliations:** 1 Department of Computer Science and Engineering, M S Ramaiah University of Applied Sciences, Bengaluru 560054, India; samahit@gmail.com; 2 Department of Oral Pathology and Microbiology, Faculty of Dental Sciences, M S Ramaiah University of Applied Sciences, Bengaluru 560054, India; drroopasrao1971@gmail.com; 3 Department of Oral Pathology, Institute of Dental Sciences, Bareilly 243006, India; madhutanu@gmail.com; 4 Department of Oral & Maxillofacial Pathology & Microbiology, Manipal College of Dental Sciences, Manipal 576104, India; chetana.c@manipal.edu (C.C.); raghu.ar@manipal.edu (R.R.); 5 Department of Oral Pathology, SVS Institute of Dental Sciences, Mahbubnagar 509001, India; sailajasanjeeva@gmail.com; 6 Department of Oral & Maxillofacial Pathology & Microbiology, Maratha Mandal’s Nathajirao G Halgekar, Institute of Dental Science & Research Centre, Belgaum 590010, India; drviju18@yahoo.com; 7 Department of Oral & Maxillofacial Pathology, Nijalingappa Institute of Dental Science & Research, Gulbarga 585105, India; munna.pmk@gmail.com

**Keywords:** CNN, deep learning, image classification, whole-slide imaging, OKC, non-KC, ReliefF

## Abstract

The microscopic diagnostic differentiation of odontogenic cysts from other cysts is intricate and may cause perplexity for both clinicians and pathologists. Of particular interest is the odontogenic keratocyst (OKC), a developmental cyst with unique histopathological and clinical characteristics. Nevertheless, what distinguishes this cyst is its aggressive nature and high tendency for recurrence. Clinicians encounter challenges in dealing with this frequently encountered jaw lesion, as there is no consensus on surgical treatment. Therefore, the accurate and early diagnosis of such cysts will benefit clinicians in terms of treatment management and spare subjects from the mental agony of suffering from aggressive OKCs, which impact their quality of life. The objective of this research is to develop an automated OKC diagnostic system that can function as a decision support tool for pathologists, whether they are working locally or remotely. This system will provide them with additional data and insights to enhance their decision-making abilities. This research aims to provide an automation pipeline to classify whole-slide images of OKCs and non-keratocysts (non-KCs: dentigerous and radicular cysts). OKC diagnosis and prognosis using the histopathological analysis of tissues using whole-slide images (WSIs) with a deep-learning approach is an emerging research area. WSIs have the unique advantage of magnifying tissues with high resolution without losing information. The contribution of this research is a novel, deep-learning-based, and efficient algorithm that reduces the trainable parameters and, in turn, the memory footprint. This is achieved using principal component analysis (PCA) and the ReliefF feature selection algorithm (ReliefF) in a convolutional neural network (CNN) named P-C-ReliefF. The proposed model reduces the trainable parameters compared to standard CNN, achieving 97% classification accuracy.

## 1. Introduction

The odontogenic keratocyst (OKC) is a developmental cyst that arises from the dental lamina or remnants of the dental epithelium. It is often classified as an odontogenic cyst due to its origin in dental tissues. The characteristic histopathological features with regard to the OKC are a stratified epithelium with 10–11 distinct layers of cells, the absence of rete ridges, a well-defined basal cell layer with cuboidal or columnar cells arranged in a palisaded fashion resembling a “picket fence” or a “tombstone appearance”, and a thin, spinous layer with surface keratin [1,2]. Radicular cysts comprise an arcading pattern of the odontogenic epithelium with inflammatory infiltrate, and dentigerous cysts possess 2–3 layers of odontogenic epithelium, representing reduced enamel epithelium. Differentiating one from another is vital, as the treatment differs. OKCs require special attention due to their inherently aggressive biological behavior, which tends to recur after surgical treatment [3,4]. Early and accurate histopathological diagnosis prevents unwanted complications, such as pathological fracture or jaw resection morbidity, and achieves better treatment outcomes [5].

A WSI, also known as a virtual slide or digital slide, is a high-resolution digital representation of an entire histopathology glass slide in gigabytes. WSIs capture the entire image in one go and allow regions of interest to be zoomed into and out of, which can be a rather tedious procedure using a microscope. The resulting digital image is a large, multi-gigapixel file that preserves all of the information present on the original glass slide. WSIs allow for various image analysis techniques, including computer-assisted algorithms for quantification and feature extraction [6].

CNNs are applied to whole-slide image (WSI) processing for several reasons, primarily due to their ability to automatically learn hierarchical features from image data. The paragraphs below explain how CNNs are applied to WSI processing and why they are used. 

Local Feature Learning: WSIs are extremely large images, often containing detailed structures and regions of interest. By applying convolutional layers, the network can automatically learn relevant local features. 

Hierarchy of Features: CNNs are designed to capture features at different levels of abstraction, from simple edges and textures to complex structures. This hierarchical feature learning is well suited for medical image analysis, where different structures (e.g., cells and tissues) can have varying levels of complexity [7,8,9].

Automatic Feature Extraction: Traditional methods for processing medical images often rely on manual feature extraction, which can be time-consuming and prone to human error. CNNs can automatically learn and extract relevant features from medical images without the need for hand-crafted features. Overall, CNNs are a powerful tool for medical image processing due to their ability to learn relevant features, handle large images, and automate complex tasks, making them an asset in medical imaging and other fields that deal with high-resolution images [10].

The objective of this research was to develop an integrated deep-learning-based model to detect OKCs with accuracy and consistency. The proposed model provides an automated diagnostic system that can serve as a decision support tool for pathologists locally or remotely, giving them more data and insights to improve their decision-making skills. This reduces healthcare costs in remote places. The contribution of this research work is that the CNN-trainable parameters are reduced significantly without compromising quality and generalization, using an integrated approach with PCA and ReliefF. This reduction in parameters gives the competitive advantages of reduced overfitting, lower memory usage, less computational power, and less time to train. Therefore, this model or system can be used in cost-effective cloud environments or resource-constrained environments.

## 2. Related Work

### 2.1. Tile Generation and Its Advantage

The act of partitioning an image into smaller sections is frequently referred to as “tiling” or “image tiling”. Image tiles can be used for various purposes, such as the following: parallel processing—by breaking an image into smaller tiles, multiple processing units or cores can work on different tiles simultaneously, speeding up the overall image processing; memory optimization—for large images, it may not be feasible to load the entire image into the memory at once, and tiling allows the processing of smaller portions of the image one at a time, reducing memory requirements; image compression and transmission—in image compression or transmission, the image can be divided into tiles, and each tile can be independently compressed or transmitted, making the process more efficient. When working with image tiles, it is essential to consider the potential impact of dividing the image, especially near the edges of objects of interest, as the tiles may not capture complete objects and context in those cases. Proper overlap or padding can be used to mitigate this issue, depending on the specific task and requirements [11,12].

### 2.2. Tile-Based CNN Processing

WSI classification was achieved by preprocessing the WSI into tiles of size 224 × 224. The authors took a specific approach to classify the slides based on the classification of the tiles. When 100% of the tiles are negative, then it considers the slide to be negative, whereas if one of them is positive, it is considered positive. The pretrained CNN model Resnet-34 was used [13].

### 2.3. CNN in WSI Classification

The first classification study using microscopic images to detect OKCs used the Google Inception v3 model. Inception v3 uses “Inception” modules, which are a combination of multiple convolutional layers of different filter sizes and pooling operations. The Inception module allowed the network to capture multi-scale patterns efficiently, enabling the model to recognize both fine-grained and large-scale features in the images. It achieved a good accuracy rate using transfer learning [14]. For panoramic radiographs, YOLOv3 was used. This model achieved an accuracy of 94 in detecting OKCs using radiographic features [15]. A patch-based hierarchical deep-learning framework that used two CNNs was used to classify WSIs based on the patch level. Patch-based classification is a technique used in computer vision and image analysis to classify images by dividing them into smaller regions or patches and then making predictions for each patch individually. This approach is commonly used in scenarios where the spatial distribution of objects or features of interest varies across the image [16]. A combination of multi-scale attention and VGG16 was used to classify slides of different resolutions. It used a bigger-tile-size image of 4096 × 4096. Subsequently, using these tiled images, it cropped the image to a smaller size of 224 × 224 to feed into the model. It used multi-scale attention to give importance to the patch that contributed most to the final classification [17]. A Python-based open slide framework was used to obtain tiles from the base level of the slide pyramid. Data augmentation techniques were applied after the selection [18] (Python 3.10).

### 2.4. Feature Extraction and Selection

CNN architecture can be used to extract features from different inputs [19]. One of the major challenges in training CNNs lies in the necessity to train a large number of parameters. In certain cases, Multilayer Extreme Learning Machines (ML-ELMs) are employed. The random feature technique was used in ML-ELMs and was also non-iterative and fast [20]. Feature selection using various feature selection methods can enhance the classification accuracy. Feature selection can be applied to select features from images or to select features generated by the pretrained CNN. Feature selection can improve the overall performance. Canonical Correlation Analysis (CCA), a multivariate-based correlation statistical method used with ReliefF and CNN, is possible with different pretrained models [21,22,23].

### 2.5. Dimensionality Reduction and Class Imbalance Problem

A combination of principal component analysis (PCA), CNN, and an attention-based algorithm was used to achieve higher classification accuracy. PCA transforms high-dimensional data into a lower-dimensional space while preserving the most important patterns and variations present in the original data. It achieves this by identifying the principal components, which are new orthogonal axes that represent the directions of maximum variance in the data [24]. The “Synthetic Minority Over-sampling Technique” (SMOTE) is a popular data augmentation method used to address class imbalance in machine learning. Class imbalance occurs when one class (the minority class) has significantly fewer samples than another (the majority class). SMOTE works by generating synthetic samples for the minority class to balance the class distribution. It does this by creating new synthetic instances that interpolate between existing instances of the minority class [25].

## 3. Materials and Methods

This section describes the complete implementation steps and the different techniques used in each step.

### 3.1. Data Collection

A multi-center study was undertaken to consolidate histopathology slides from various centers in India that volunteered to take part in the research: the Manipal College of Dental Sciences (MCODS), Manipal; the Institute of Dental Science, Bareilly; S Nijalingappa Institute of Dental Sciences and Research, Rajapur, Kalaburagi, Karnataka; Maratha Mandala Dental College, Belagavi, Karnataka; and the SVS Institute of Dental Sciences, Mahbubnagar, Andhra Pradesh. The institute permitted this, as the study involved archived slides (2014–2021) and received ethics clearance (No. EC-2021/F/058) from MS Ramaiah University of Applied Sciences. Ethics approval was waived for slides collected from diverse centers due to the study’s retrospective nature. Furthermore, the slides were blinded and coded without any patient identities. Whole-slide images were captured by Morphle Labs Whole Slide Scanner Model-Index.

Of 113 archival specimens in total, 48 OKC, 20 dentigerous cyst (DC), and 37 radicular cyst (RC) whole-slide images were collected at 40× magnification. These slides varied in size from 50 megabytes to 3 gigabytes depending on the region selected for scanning to obtain the whole-slide image.

### 3.2. Data Preprocessing and Dataset Generation

These whole-slide images were inspected by an experienced pathologist and categorized into three types: odontogenic keratocyst, dentigerous cyst, and radicular cyst. These slides were manually labeled by an experienced pathologist.

**Figure 1:**
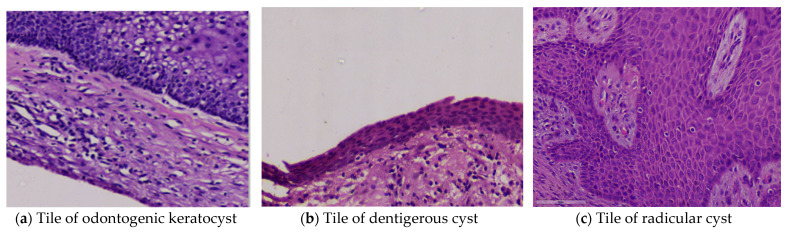
Tile images (**a**–**c**).

**Figure 2:**
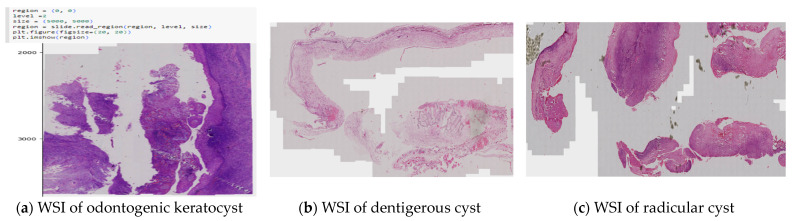
Whole-slide images (**a**–**c**).

The slides were processed through an automated pipeline system developed using a deep zoom generator and open slide library in Python to generate tiles of 2048 × 2048 size, as shown in Figure 1 and Figure 2. This automation pipeline efficiently removes white tiles and those with minimal or non-important information. This was achieved using the OTSU threshold technique. Any blurry tiles or those that were too dark were manually segregated and omitted from consideration. 

**Figure 3:**
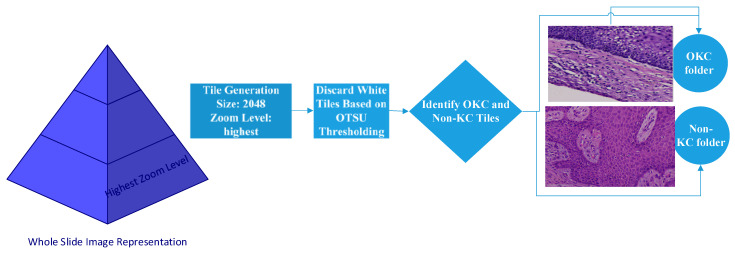
Tile generation flow diagram.

In this step, 48 OKC slides, 20 DC slides, and 37 RC slides were selected for the entire tile dataset creation, as detailed in Table 1 and Table 2. Non-KC WSIs had a pyramid structure with many resolution levels. In this case, the highest zoom level was taken for each slide for generating the tiles, as a low resolution may impact the performance of the model. Hence, tiles generated from the base-level slide, which had the most prominent features of OKCs and non-KCs, were fed into the classification pipeline. In our dataset, we took 48 OKC WSIs, generated 6069 positive labels (OKC), and considered 57 (DC and RC) WSIs, which generated 5967 tiles with negative labels. The dataset preparation flow is given in Figure 3. The difference between the number of images in the two classes was nearly 1%; hence, there was no class imbalance issue to address and no need for oversampling or undersampling to balance the dataset.

## 4. Methodology

### 4.1. Proposed Novel Tile Classification Algorithm

The tile dataset developed as part of the preprocessing step was considered for training purposes. The Keras train generator augmentation technique (rotation range = 20; width_shift_range = 0.1; height_shift_range = 0.1; shear range = 0.2; zoom_range = 0.2; horizontal_flip = True) was used to process the image, which was then fed to the proposed model for training and validation. The images were resized to 64 × 64 and then fed into the model for training.

**Figure 4:**
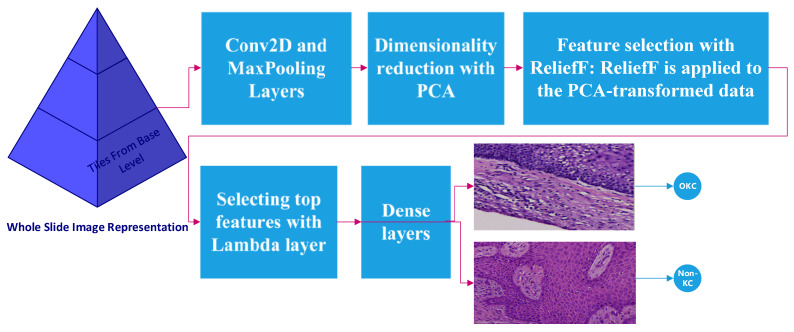
Flow diagram for proposed P-C-ReliefF algorithm.

The 80–20 rule was followed for training and validation. All of the training and validation data for non-KC tiles were taken only from DC and RC WSIs. Figure 4 represents the flow of the algorithm.

### 4.2. P-C-ReliefF Architecture and Parameters

**Figure 5:**
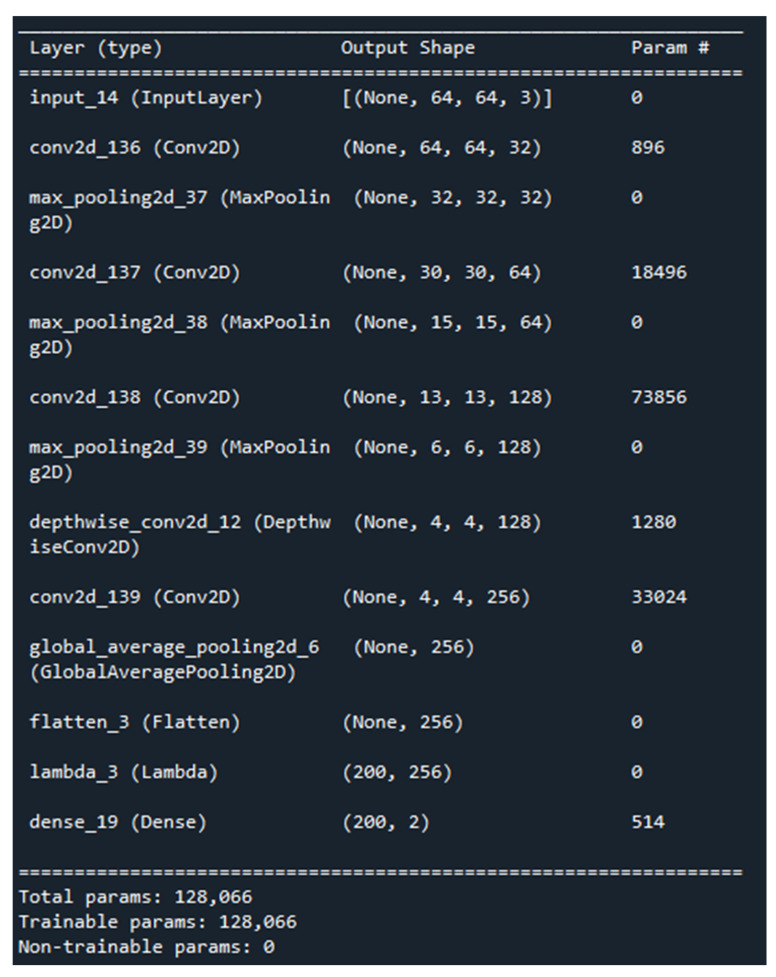
P-C-ReliefF model summary with parameters.

**Figure 6:**
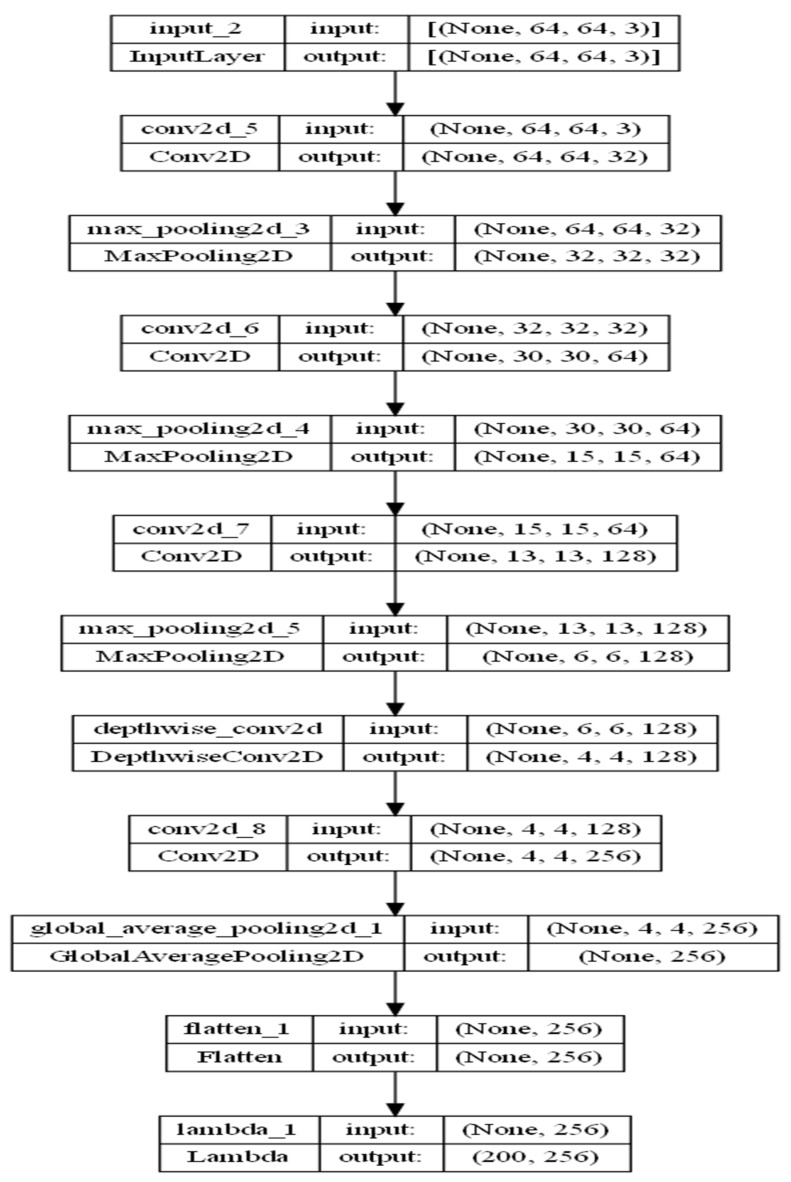
P-C-ReliefF model architecture.

The model architecture was defined using the Keras functional API. The P-C-ReliefF summary with parameters and architecture are shown in Figure 5 and Figure 6. The process started with a series of Conv2D layers with varying parameters and activation functions, followed by MaxPooling2D layers for downsampling. The architecture also included a DepthwiseConv2D layer and a subsequent Conv2D layer. GlobalAveragePooling2D was used to aggregate the spatial information, and flattening was applied to obtain a 1D representation (CNN features) from the output tensor.

Dimensionality reduction with PCA: The flattened output tensor was the input data, and it was reshaped to match the required format for applying PCA. PCA was then performed on the training data to reduce the dimensionality to a specified number of components.

Feature selection with ReliefF: ReliefF was applied to the PCA-transformed data to rank the features based on their relevance to the target labels. ReliefF is a popular feature selection algorithm used for machine-learning tasks, particularly in the context of classification problems.

Key definitions and concepts related to the ReliefF algorithm: Feature relevance: ReliefF aims to estimate the relevance (importance) of each feature concerning the target class labels. It measures how well a feature discriminates between different classes. Feature weight: Each feature is assigned a weight representing its relevance. A higher weight indicates a more important feature for classification. Feature scores: The feature relevance scores, also known as feature weights or feature importance, are typically represented as a vector of real numbers, with each element corresponding to a feature.

Nearest neighbor (NN): ReliefF is based on the idea of comparing the feature values with those of the nearest neighbors of each instance. The nearest neighbor is typically defined by some distance metric (e.g., Euclidean distance). Hit and miss: For each instance in the dataset, ReliefF distinguishes between features that are like the nearest neighbor of the same class (hit) and features that are like the nearest neighbor of a different class (miss).

Weight updating: The weights of the features are updated based on the differences between hits and misses. If a feature’s value is close to the corresponding feature value of the nearest neighbor of the same class (hit), its weight is increased. If the feature’s value is close to the nearest neighbor of a different class (miss), its weight is decreased. Multiple Iterations: ReliefF usually performs multiple iterations over the dataset to improve the estimation of feature relevance. During each iteration, the nearest neighbors of instances are updated.

Efficiency: ReliefF is computationally efficient in handling datasets with many instances and features. ReliefF is a widely used feature selection method because it is relatively simple to implement, performs well on a variety of datasets, and provides insights into the importance of features for classification tasks. It helps to identify relevant features, leading to improved model performance and reduced overfitting, especially when dealing with high-dimensional datasets.

Selecting top features with Lambda layer: In general, a Lambda layer is a custom layer in a deep-learning model that allows one to apply arbitrary transformations to the input data during the forward pass (also known as the forward propagation). It provides a way to include user-defined functions or operations that are not directly available as built-in layers in the deep-learning framework. The Lambda layer is available in various deep-learning frameworks, such as Keras and TensorFlow. In Keras (which is now part of TensorFlow), it is specifically named Lambda. Key points about the Lambda layer: Custom transformations: The Lambda layer allows one to define a function (or Lambda function) that operates on the input tensor and returns the transformed output tensor. This function can include any operations that the user wants to apply to the input data, such as mathematical computations, reshaping, slicing, or other custom operations. Flexibility: The Lambda layer gives the flexibility to integrate custom logic into the model without needing to create a new layer from scratch. It can be particularly useful when there is a need to perform specific data manipulations that are not readily available as standard layer types. The CNN features were selected based on the sorted importance scores (sorted indices) using a Lambda layer and the custom select feature function. The selected features were stored in selected features. Additional dense layers: Two dense layers were added after the selected features, with the specified number of units and activation functions. The models use the Adam optimizer, binary cross-entropy loss, and accuracy as the evaluation metric. Model summary: The model summaries were printed to provide an overview of the model architecture, including the layers, output shapes, and parameter counts.

## 5. Results

The experiment involved standard CNNs with similar architecture and 1,700,161 standard model CNN-generated parameters. A comparison of the model parameters and hyperparameters was carried out, as shown in Table 3 and Table 4. VGG16 had 14,846,273 parameters, and VGG19 had 20,155,969 parameters, whereas our P-C-ReliefF only generated 128,066 parameters. In between the two CNN layers, two depthwise convolution networks were used. Depthwise convolution is a type of convolutional layer used in deep-learning models, especially in mobile and resource-constrained architectures. Unlike traditional convolutions that apply filters across all input channels (also known as “spatial” dimensions), depthwise convolution applies separate filters for each input channel independently. This helped to reduce more than 95% of the trainable parameters after multiple experiments on both depthwise and pointwise convolution layers.

### 5.1. Confusion Matrix

**Figure 7:**
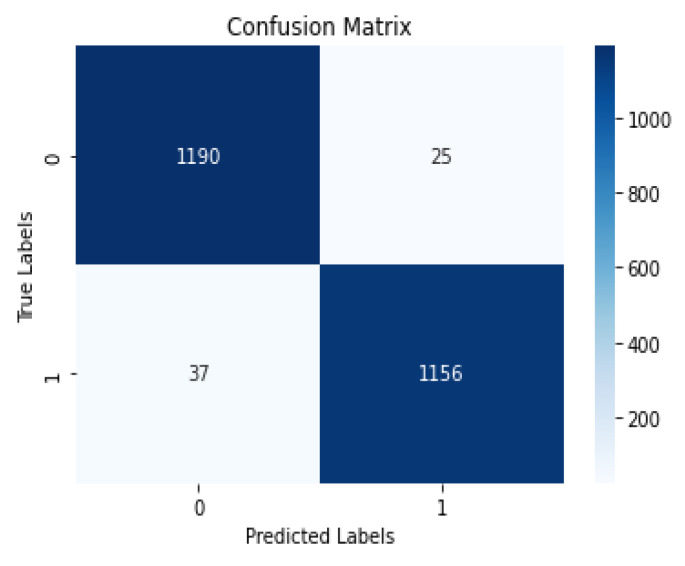
P-C-ReliefF confusion matrix.

A confusion matrix is a 2 × 2 table used in classification tasks to assess the performance of a machine-learning model, as shown in Figure 7.

### 5.2. ROC Curve

**Figure 8:**
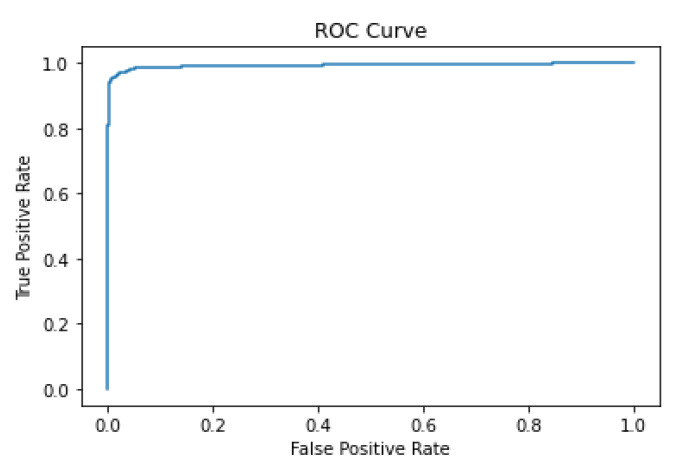
P-C-ReliefF ROC curve.

The AUC score is a useful metric in situations where class imbalances exist in the dataset, as it assesses the classifier’s performance irrespective of the decision threshold. It also provides a single value to compare different classifiers’ performance, making it easier to evaluate and choose the best model for a given task. The score of 0.97 indicates that the classifier’s performance is better than the standard CNN at 0.93, as shown in Figure 8. The ROC (receiver operating characteristic) is used to evaluate the performance of binary classification models. It plots the true-positive rate (sensitivity) against the false-positive rate (1-specificity) at various classification thresholds. The ROC curve illustrates how well the model distinguishes between positive and negative classes, with the ideal model’s curve reaching the upper-left corner. The area under the ROC curve (AUC) is a common metric; a higher AUC indicates better model discrimination ability.

### 5.3. Training vs. Validation Loss Curve

**Figure 9:**
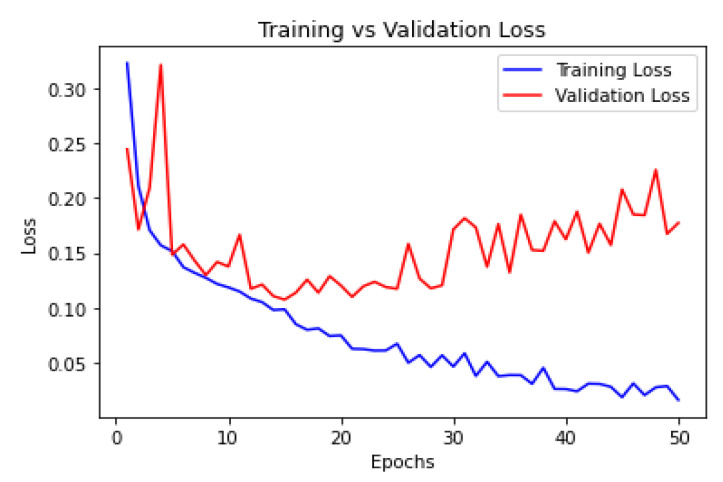
P-C-ReliefF.

The training vs. validation loss curve is a plot that shows the changes in the training and validation loss during the training process of a machine-learning or deep-learning model, as shown in Figure 9.

The training vs. validation loss curve was plotted with the epochs (training iterations) on the *x*-axis and the corresponding loss values on the *y*-axis. As the model was trained over multiple epochs, the training loss generally decreased because the model was learning to fit the training data better. However, the validation loss may behave differently. Initially, it decreased along with the training loss as the model generalized better. However, at some point, the validation loss may start to increase. This indicates that the model was overfitting the training data, and its performance on the validation data deteriorated, even though it improved on the training data.

### 5.4. Classification Report: PCNN-ReliefF

**Figure 10:**
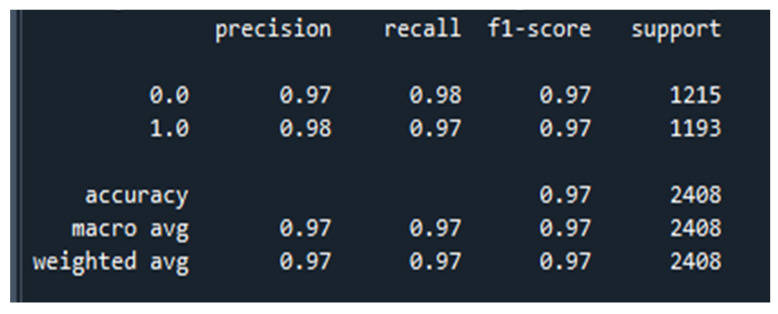
P-C-ReliefF classification report.

A classification report is a summary of performance metrics for a classification model, typically presented in a tabular format. It includes key metrics such as precision, recall, F1-score, and support for each class in a multi-class classification problem, as shown in Figure 10. This report provides insights into the model’s performance for individual classes, highlighting the strengths and weaknesses. It is a valuable tool for evaluating the effectiveness of a classification model across different categories.

### 5.5. Log Loss

Log loss (logarithmic loss) is a commonly used loss function for evaluating the accuracy of probabilistic classification models, such as logistic regression or neural networks, that predict probabilities for each class. It measures the discrepancy between predicted probabilities and actual target values, penalizing larger deviations, as shown in Table 5. Lower log-loss values indicate better alignment between predicted probabilities and true outcomes.

The proposed model has a log-loss value of 0.129, indicating that the model’s predicted probabilities are quite accurate and very close to the true labels. In binary classification, a log loss close to zero indicates excellent performance, as it means that the model’s predicted probabilities align well with the actual outcomes.

A standard log loss value of 1.390 means that, on average, the model’s predicted probabilities are not very close to the true labels. It suggests that the model’s confidence in its predictions might be relatively low or that there is room for improvement in the model’s calibration.

### 5.6. Other Metrics for Proposed Model

The performance of the proposed model is shown in Table 6. These are the commonly used metrics for a classification problem.

## 6. Pipeline Result

**Figure 11:**
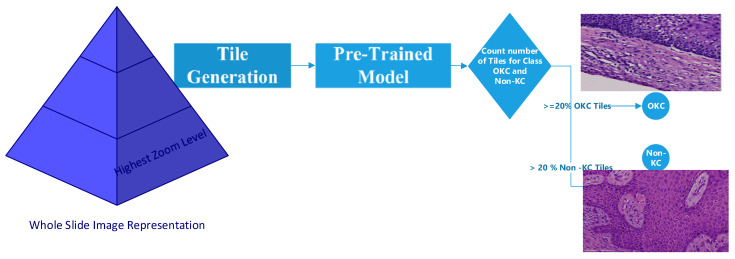
Workflow diagram of pipeline WSI prediction.

A pipeline system was designed to predict the entire WSI as OKC or non-KC. This pipeline system was the integration of the tile generation workflow, with each tile being fed into a pretrained model. This pretrained model will predict the correct label of the tile. Based on the number of tiles predicted in both classes, a threshold was designed. This threshold was set based on the size and zoom level of the slide. Most of the time, a 20% threshold works; however, for some smaller-sized slides, the threshold can be lower. It was set based on the decision of the pathologist who was familiar with the whole-slide scanner. Overall, the model was applied to 10 different slides, and the statistics are shown in Figure 11 and Table 7.

## 7. Discussion

ReliefF feature selection was performed, along with obtaining the feature importance, sorting the importance, and using a custom Lambda layer to select the top features from the CNN layer features. The selected features were then used to train a separate classifier model. The feature selection technique used was ReliefF, which is a filter-based feature selection method. It selects a subset of features based on their importance scores without modifying the original model architecture or the number of trainable parameters. After applying the ReliefF feature selection, the selected features are used as inputs to a separate classifier model.

The original CNN layers remain unchanged, including their architecture and the number of trainable parameters. ReliefF feature selection only affects the input to the classifier model, not the CNN layers themselves. By selecting a subset of important features, ReliefF aims to improve the performance of the classifier by reducing the potential noise or irrelevant information present in the original features. However, it does not directly reduce the number of trainable parameters in the CNN layers.

Dimensionality Reduction: Selecting a subset of features reduces the dimensionality of the input space. This can help reduce computational complexity and potential overfitting, especially when dealing with high-dimensional data. Generalization: By selecting the most relevant features, the model may generalize better to unseen data. The selected features were expected to capture the underlying patterns and variations in the data, leading to better generalization performance. Overall, feature selection can help improve the efficiency, interpretability, and generalization ability of the model by selecting the most informative features. It allows the subsequent dense layer to focus on the most relevant aspects of the data, potentially leading to improved classification performance.

The objective was to significantly reduce the number of parameters while improving the model’s performance compared to state-of-the-art models, as shown in the different experiments in Table 3. The log-loss value of the proposed model was lower as compared to the standard CNN. Another advantage is the improved generalization of the model by reducing the trainable parameters using feature selection after the CNN layer. OKCs have very distinct features, for which feature selection helped the overall accuracy with fewer parameters.

## 8. Conclusions

In the proposed model, the trainable parameters were reduced significantly without compromising accuracy; moreover, the training and validation time were also reduced significantly with this model. OKCs and non-KCs have distinguishing features, and the feature selection algorithm helps to reduce the complexity of dense layers for classifying OKCs correctly. ReliefF feature selection is a popular feature selection technique with proven results. Hence, this model’s behavior in automating pipelines is consistent in the detection of OKCs or non-KCs. During different iterations, several experiments were conducted to adjust the threshold over a number of tiled images with OKC features, and a threshold of between 15 and 25% of the tiled images were detected in the WSI based on the size or zoom level. This pipeline helps pathologists to segregate OKCs and non-KCs locally or remotely. Even if no expert pathologist is available remotely, this pipeline can help to manage OKCs efficiently.

## 9. Drawbacks

Although the proposed model’s accuracy is very high, its accuracy in detecting OKCs can still be improved. When building the dataset, artifacts such as blurriness, too much color variation, and poor quality of the slides were excluded for tile generation. This means that the model requires perfect slides to diagnose OKCs correctly. Hence, research in this area can be suitably extended by considering these slides with the help of expert pathologists. The tile-based approach needs significant time initially to label each tile based on the distinct features of OKCs. Since OKCs have distinct histological features visible on high-resolution images, it was easy, but time-consuming, for the pathologist to label the tiles. However, this research can be suitably extended to other whole-slide image problems by suitably tuning the required model parameters. The proposed method can be further strengthened by extending the model by integrating it with the vision transformer method.

## Figures and Tables

**Table 1:** OKC WSIs and statistics of generated tiles.

Total Number of WSIs Labeled as OKC	Total Number of White and Non-Important Information Tiles (Discarded)	Total Number of Tiles Considered for Analysis of OKC	Total Number of Tiles Labeled as OKC (Tiles with Epithelium Layer) by Pathologist for Training and Validation
48	111,600	42,000	6069

**Table 2:** Non-KC WSIs and statistics of generated tiles.

Total Number of WSIs Labeled as Non-KC	Total Number of White Tiles (Discarded)	Total Number of Tiles Labeled as Non-KC for Training and Validation
20 (DC)	53,000	2548
37 (RC)	104,500	3419

**Table 3:** Model performance metrics and parameter comparison.

Model	Recall	Precision	F1-Score	AUC	Accuracy	Total Parameters
Standard CNN	0.96	0.96	0.96	0.93	0.96	1,700,161
VGG19	0.96	0.97	0.96	0.93	0.96	20,155,969
VGG16	0.97	0.97	0.97	0.93	0.97	14,846,273
Inception V3	0.96	0.96	0.95	0.95	0.96	23,901,985
P-C-ReliefF (Proposed Method)	0.98	0.98	0.98	0.99	0.97	128,066

**Table 4:** Model hyperparameters.

Component	CNN	P-C-ReliefF
Learning Rate	0.001	0.001
PCA Components + ReliefF Top Features	NA	200
Number of Model Parameters	1,700,161	128,066
Loss Function	Binary cross-entropy	Binary cross-entropy
Optimizer	Adam optimizer	Adam optimizer

**Table 5:** Log loss comparison.

Standard CNN	P-C-ReliefF
1.390	0.129

**Table 6:** Performance metrics.

Metrics	Value
Accuracy	0.974
Precision	0.979
Recall	0.979
F1-Score	0.975
Matthews Correlation Coefficient	0.949
Cohen’s Kappa	0.948
Balanced Accuracy	0.974
Jaccard Score	0.949
Brier Score Loss	0.025
Specificity (True Negative Rate)	0.979
Sensitivity (True Positive Rate)	0.969
Youden’s Index (J)	0.948
G-Mean	0.974
Log Loss	0.129
Validation Loss	0.177

**Table 7:** Sample statistics of pipeline result.

Case No.	File Size (MB)	Base Resolution (H, W)	No. of Tiles	No. of OKC Tiles	No. of Non-KC Tiles	Predicted Output	Actual Output
375_68_22	658	126,976 × 126,976	3844	968	2876	OKC	OKC
275_149_20	179	126,976 × 31,744	992	50	942	Non-KC	Non-KC

## Data Availability

Not applicable.
